# Exosome removal as a therapeutic adjuvant in cancer

**DOI:** 10.1186/1479-5876-10-134

**Published:** 2012-06-27

**Authors:** Annette M Marleau, Chien-Shing Chen, James A Joyce, Richard H Tullis

**Affiliations:** 1 Aethlon Medical Inc, 8910 University Center Lane, Suite 660, San Diego, CA, 92122, USA; 2 Division of Hematology/Oncology, Loma Linda University School of Medicine, 11175 Campus Street, Chan Shun Pavilion, 11015, Loma Linda, CA, 92354, USA

**Keywords:** Exosomes, Cancer, Immune suppression, Metastasis, Affinity agents, Plasmapheresis cartridges, Dialysis, Lectin, Antibodies

## Abstract

Exosome secretion is a notable feature of malignancy owing to the roles of these nanoparticles in cancer growth, immune suppression, tumor angiogenesis and therapeutic resistance. Exosomes are 30–100 nm membrane vesicles released by many cells types during normal physiological processes. Tumors aberrantly secrete large quantities of exosomes that transport oncoproteins and immune suppressive molecules to support tumor growth and metastasis. The role of exosomes in intercellular signaling is exemplified by human epidermal growth factor receptor type 2 (HER2) over-expressing breast cancer, where exosomes with the HER2 oncoprotein stimulate tumor growth and interfere with the activity of the therapeutic antibody Herceptin®. Since numerous observations from experimental model systems point toward an important clinical impact of exosomes in cancer, several pharmacological strategies have been proposed for targeting their malignant activities. We also propose a novel device strategy involving extracorporeal hemofiltration of exosomes from the entire circulatory system using an affinity plasmapheresis platform known as the Aethlon ADAPT™ (adaptive dialysis-like affinity platform technology) system, which would overcome the risks of toxicity and drug interactions posed by pharmacological approaches. This technology allows affinity agents, including exosome-binding lectins and antibodies, to be immobilized in the outer-capillary space of plasma filtration membranes that integrate into existing kidney dialysis systems. Device therapies that evolve from this platform allow rapid extracorporeal capture and selective retention of target particles < 200 nm from the entire circulatory system. This strategy is supported by clinical experience in hepatitis C virus-infected patients using an ADAPT™ device, the Hemopurifier®, to reduce the systemic load of virions having similar sizes and glycosylated surfaces as cancer exosomes. This review discusses the possible therapeutic approaches for targeting immune suppressive exosomes in cancer patients, and the anticipated significance of these strategies for reversing immune dysfunction and improving responses to standard of care treatments.

## Tumor-derived exosomes as biologic messengers in cancer

A large body of literature has documented the relationship between suppressed immune status and cancer progression resulting from tumor-mediated mechanisms as well as from immune ablation caused by the therapeutic agents themselves [1]. Although clinical trials have tested a plethora of vaccination approaches against cancer, tumor regression has been difficult to achieve and reversal of the immune dysfunction in cancer patients remains an important therapeutic goal. The possibility of utilizing immune “de-repressive” approaches to augment the efficacy of existing therapies is enticing; therefore, there is a need to identify the appropriate targets and develop avenues for interfering with their activity.

Numerous studies have shown that exosomes secreted by tumor cells serve as vehicles for immune suppression and other pro-cancer activities. Exosomes are one of a heterogeneous group of microvesicles, distinguished by their small size (30–100 nm) and cup shaped morphology, that are secreted by a variety of cell types under physiological and pathological conditions [2]. Exosome biogenesis begins with endosomes that form in clathrin-coated vesicles at the plasma membrane, which are enriched for integral membrane proteins [3]. The molecules that are present in endosomes can either be recycle back to the plasma membrane or become incorporated into intralumenal vesicles (ILV). These vesicles accumulate in maturing endosomes by inward budding of the endosomal membrane, thereby transforming endosomes into multivesicular bodies (MVB). The sorting of cargo into ILV is mediated by a protein complex known as the ESCRT (endosomal sorting complexes required for transport) machinery that recognizes ubiquitinated proteins and facilitates their inclusion into ILV of MVB [4]. Subsequently, MVB either fuse with lysosomes where their contents are degraded or they fuse with the plasma membrane and expel their internal vesicles, known as exosomes, into the extracellular space through outward budding from the membrane [5]. Tumor-derived exosomes are released locally and into the circulation to interact with a variety of target cells, including other tumor cells, endothelial cells and immune cells, which occurs via uptake of the exosomes by endocytosis, direct plasma membrane fusion, or receptor-mediated adhesion to target cells [6]. The vacuolar H^+^-ATPase transmembrane pumps that maintain the low pH of the tumor microenvironment are essential for fusion of tumor-derived exosomes with target cells, which is believed to be related to higher rigidity of exosomal membranes at a lower pH [7]. Interestingly, one study revealed that exosome secretion is induced by detachment of breast cancer cells from substrata, and these exosomes subsequently accumulate in lipid raft domains on the cell surface for adhesion and spreading of tumor cells [8].

The ability of exosomes to serve as purveyors of long distance signals between cells is facilitated by their double-layer membrane enriched in cholesterol, sphingomyelin, and ganglioside GM3 as well as by protective proteins against complement, thereby allowing for a stable conformation and superior biodistribution of their protein repertoire in comparison to free-floating proteins [5]. All exosomes, irrespective of their cell type of origin, contain a conserved set of proteins involved in cell adhesion, cell structure, membrane fusion, metabolism, and signal transduction [9]. Since exosomes also contain cell-type specific proteins and genetic material from their parental cells, the enrichment of tumor-secreted exosomes for factors that promote malignancy is being explored as a prognostic indicator of advancing malignancy in several types of cancer [9]. In samples of body fluids from cancer patients, including blood from breast cancer [10], ovarian cancer [11], and glioblastoma patients [12], and in urine samples from patients with prostate cancer [13,14], cancer-specific proteins and microRNA signatures in exosomes were found to serve as biomarkers of tumor type and stage. Patients with melanoma, lung cancer and gynecological cancers have higher levels of circulating exosomes compared to healthy subjects, and the concentrations of exosomes correlate with the malignant behavior of the cancer [15-18]. In a study of ovarian and endometrial cancer, microvesicles from patients with advanced cancer were found to contain matrix metalloproteinases and FasL, which have roles in cancer cell invasion and killing of immune cells, respectively, whereas these microvesicles were not detected in sera from healthy control subjects or patients with benign disease [17].

## Exosomes as mediators of tolerance induction

Immunological functions of exosomes were first identified in B cells through studies demonstrating that these cells contain a late endocytic compartment, called MIIC [major histocompatibility complex (MHC) class II-enriched compartment], that harbors newly synthesized MHC class II molecules in transit to the plasma membrane [19]. It was demonstrated that the MIIC compartment fuses with the plasma membrane, leading to the release of vesicles that display MHC class II molecules and are capable of stimulating antigen-specific T cell responses *in vitro* as well as *in vivo*[20]. These vesicles were termed “exosomes” in reference to the original work on reticulocytes [21]. A plethora of immune stimulatory roles for exosomes have been uncovered, including exosome-mediated promotion of T cell-mediated autoimmunity [22] and induction of immune responses directed against intracellular pathogens [23-25]. Moreover, exosomes derived from tumor antigen-loaded dendritic cells (DC) could be exploited as cell-free cancer vaccines, owing to their display of MHC/peptide complexes and their capacity to stimulate NK cell- and T cell responses in experimental animals and cancer patients [26-29].

The discovery that exosomal cargo mirrors that of their originating cell types has lead to the understanding that exosomes can be either immune stimulatory or tolerogenic depending on the originating cell’s activation state and the cellular cargo that is packaged into the vesicles. DC are key orchestrators in whether immune activation or immune tolerance occurs as a result of their interactions with T cells. It has been reported that immature DC promote induction of tolerance [30], and that administration of these “tolerogenic DC” can suppress autoimmunity *in vivo*[31]. Given observations that exosomes derived from mature DC are immune stimulatory [32,33], the theory was tested that exosomes secreted by tolerogenic dendritic cells could serve as vehicles for suppressing inflammatory responses. Indeed, Ruffner et al. showed that dendritic cells treated with IL-10 to block their maturation secrete exosomes that inhibit delayed-type hypersensitivity reactions in an antigen-specific manner, an effect requiring CD80 and CD86 co-stimulatory molecules on exosomes, possibly for direct interactions with T cells [34]. Similarly, Yang et al. used donor-strain derived exosomes from immature DC to enhance intestinal allograft survival in a rat transplantation model [35]. This group demonstrated that as little as 20 μg of donor- (but not recipient-) derived exosomes were capable of significantly prolonging graft survival. Prolongation of graft survival with exosomes from immature DC was also observed in a cardiac allograft model [36]. Kim et al. demonstrated that the exosomes produced by tolerogenic DC were on average 75 nm in size and mediated their suppressive effects on T cells through their display of Fas ligand [37]. Although exosome production by tolerogenic DC propagated *in vitro* could be considered artefactual, there are also many naturally occurring examples of exosomes as mediators of immune tolerance.

Pregnancy represents an *in vivo* example where exosomes promote immune tolerance to the “fetal allograft”. During pregnancy, local and systemic immune deviation occurs [38] and the failure to induce this “natural immune modulation” is associated with recurrent spontaneous abortions [39,40]. Interestingly, exosome production has a role in directing the maternal immune system to accommodate the allogeneic fetus. Frängsmyr et al. reported that freshly isolated fetal syncytiotrophoblast cells store Fas ligand (fasL) in cytoplasmic granules that are released as exosomes, likely for the purpose of inducing apoptosis of fetus-sensitized effector T cells expressing fas [41]. As discussed in the context of dendritic cell-derived exosomes, FasL on these microvesicles is associated maintaining a state of immune privilege or tolerance. It is also plausible that exosomes bearing antigen/MHC complexes transmit death signals that cause specific killing of the T cell clones that pose a threat to the exosome-producing cell. This functional association was explored by Dr. Douglas Taylor’s group who observed that pre-term deliveries are associated with higher degrees of maternal anti-fetal immunity, as measured by TCR-zeta chain activity, and lower concentrations of FasL + exosomes [42].

Pregnancy-associated exosomes possess multiple means for modulating T cell responses. For example, the inhibitory molecule PD-1 ligand is found on pregnancy-derived exosomes in circulation, and can inhibit both CD4+ and CD8+ T cells [43]. Exosomes released by the syncytiotrophoblast of the human placenta are potently inhibitory toward maternal NK cells, CD8+ T cells, and gamma delta T cells through their expression of MHC class I chain-related proteins A and B (MICA/B), and UL-16 binding proteins (UL-BP), which are a family of ligands that bind to the natural killer activating receptor NKG2D [44,45]. Interestingly, pregnant women exhibit substantially lower expression of NKG2D on their lymphocytes as compared to non-pregnant women [45]. Culture of peripheral blood mononuclear cells from non-pregnant women with exosomes from pregnant women resulted in downregulation of NKG2D expression and suppressed NK cell activity. The immune tolerance that occurs during pregnancy has been associated with remission of autoimmunity in clinical cases of rheumatoid arthritis [46] and multiple sclerosis [47], a phenomenon that has been suggested to involve pregnancy-associated exosomes that suppress T cell responses systemically [48].

Another physiological example of exosome-mediated immune tolerance is the antigen-specific immune modulation that can be elicited in response to oral antigen administration. Induction of oral tolerance is associated with the generation of T regulatory (Treg)/Th3 cells with specificity for food-borne antigens [49]. Clinical trials of oral tolerance in rheumatoid arthritis [50], and multiple sclerosis [51,52], have shown some promising results, although the efficacy of these treatments has not met the bar for clinical approval. It was demonstrated that subsequent to feeding with a nominal antigen, plasma-circulating exosomes containing MHC II and the specific antigen could be isolated [53]. These exosomes, termed “tolerosomes”, originate from intestinal epithelial cells and engage in MHC-restricted interactions with CD4+ T cells that suppress immunological effector responses in response to the fed antigen [54]. In a murine allergy model, protection from allergy could be transferred via exosomes collected from mice that had been fed the allergen orally [55]. These data suggest that tolerance induction may occur through the generation of exosomes, as also observed for pregnancy- and cancer-associated exosomes.

## Tolerogenic functions of cancer exosomes contribute to immune evasion

**Figure 1:**
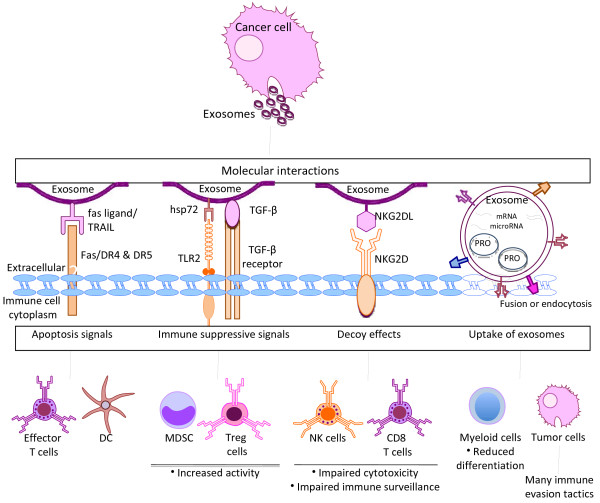
**Mechanisms of immune tolerance mediated by tumor-derived exosomes.** Exosomes evoke numerous immune suppressive pathways during their interactions with immune cells. Depicted are examples of immune suppressive interactions between tumor-derived exosomes and immune cells and their downstream effects on specific immune functions. Examples of direct adhesion and signaling interactions between surface-expressed proteins on immune cells are depicted, whereby exosomes elicit apoptosis signaling, induction of immune suppressive activity, and blockade of receptors/ligands required for anti-cancer immunity. Alternatively, exosomes and/or their contents, including proteins (PRO) and genetic material (mRNA and microRNA), are delivered directly into target cells via exosomal fusion with the target cell membrane or endocytosis. Cells that reportedly take up exosomes include immune cells (example shown) and tumor cells, which are endowed with the ability to evade immune responses through the horizontal transfer of exosomal cargo.

Many of the tolerogenic effects of exosomes secreted by healthy cells are also imparted by tumor-derived exosomes, as represented in Figure 1 and described below. The interactions between tumor-derived exosomes and immune cells are mediated through direct signaling interactions via surface-expressed molecules or by transfer of exosomes and/or their cargo to immune cells (Figure. 1). Exosomes also transport mRNAs and microRNAs to target cells, allowing for the direct exchange of genetic material originating from tumor cells [56]. Many of the signals delivered to immune cells via cancer exosomes are involved in directing the immune system to specifically ignore cancer cells. At the level of T cell immunity, exosomes possess enzymatic activity that causes hydrolysis of ATP into adenosine in the tumor microenvironment, which negatively regulates T cell activation [57]. Surface display of FasL and TRAIL on microvesicles directly engages the corresponding receptors on CD8+ T cells to induce apoptosis [58-62]. Clinical consequences of FasL on exosomes are suggested by observations that the ability of purified MAGE3/6+ (tumor antigen) FasL + microvesicles to induce T cell apoptosis *in vitro* correlates with disease activity and lymph node metastasis in head and neck cancer patients [63]. FasL on the surfaces of tumor-derived exosomes mediates cleavage of the TCR-zeta chain, a crucial T cell signaling molecule that is required for activation [64,65]. Low expression levels of TCR-zeta chain correlate with impaired immune responses and are predictive of poor prognosis of patients with several types of cancer [66-71].

Tumor-derived exosomes also promote antigen non-specific immune suppression through their effects on myeloid-derived suppressor cells (MDSC), a population of immature myeloid cells that are among the major inhibitors of T cell activation in cancer [72]. Accordingly, increased frequencies of MDSC are often detectable in the circulation of cancer patients [73,74]. Tumor-derived exosomes direct the differentiation of bone marrow myeloid progenitors to MDSC through their expression of an array of bioactive molecules, including PGE2 and TGF-β [75]. Interestingly, there is also a correlation between cancer progression and increased packaging of PGE2 and TGF-β into exosomes, which could contribute to the increased immune suppressive properties of growing tumors [75]. Cancer exosomes expressing Hsp72 also stimulate toll-like receptor 2 (TLR2) on MDSC, causing increased MDSC-mediated immune suppressive activity against T cells *in vitro*[76]. Collectively, these lines of evidence suggest that cancer exosomes increase the numbers and activity of immune suppressive cell populations.

The effects of cancer exosomes on the myeloid lineage also extend to maintaining immaturity of DC, which is associated with cancer progression in tumor-bearing hosts [77]. A study of ovarian cancer exosomes harvested from ascites fluid demonstrated their ability to induce apoptosis of DC through a Fas ligand-dependent mechanism [61]. Exosomes from human breast cancer cells inhibit the differentiation of monocytes into DC *in vitro*[78]. Similarly, microvesicles from the plasma of advanced melanoma patients, but not from healthy donors, promote the differentiation of monocytes with TGF-β-secreting activity that suppressed T cell activation and cytolytic activity [79]. Aberrantly elevated levels of TGF-β in cancer serve to increase the activity of Treg cells that promote immune suppression [80]. Additionally, tumor-derived exosomes also display TGF-β on their surfaces, which maintain the numbers and immune suppressive effects of Treg cells *in vitro*[81]. Whiteside’s group reported that tumor-derived microvesicles induce the expansion of CD4+ CD25+ FoxP3+ cells while inducing apoptosis of tumor-reactive CD8+ T cells [82,83].

NK cells play a critical role in tumor immune surveillance, as exemplified by a study that showed a higher incidence of spontaneous tumors in mice deficient in NKG2D [84], an activating immune receptor that is expressed by cytotoxic cells, including NK cells and CD8+ T cells [85]. Ligands for NKG2D are generally only expressed during cellular stress such as the DNA damage response that is initiated in response to oncogene expression [86,87]. In addition to expressing NKGD ligands, tumors also shed soluble ligands that cause downregulation of the corresponding receptor on immune cells, thereby impairing their recognition of neoplastic cells [88]. Tumor-derived exosomes display NKG2D ligands, including MICA/B, ULBP1 and ULBP2, which mask NKG2D and mediate downregulation of this receptor on NK cells and CD8+ T cells [89-92]. TGF-β1 expression by exosomes [90] also contributes to NKG2D downregulation and impaired NK cell function in cancer patients [93]. Notably, the exosomal form of NKG2D is more effective at suppressing immune cells than the soluble form since the former allows for proper orientation and biodistribution of NKG2D ligands in a stable conformational arrangement [94].

## Cancer exosomes spread tumor growth signals that counteract the activity of therapeutic agents

Exosomes have emerged as major players in transporting soluble proteins involved in cancer growth, including members of the human epidermal receptor (HER) family, which are constitutively active in many cancers as a result of gene amplification, protein over-expression, and/or mutations of their tyrosine kinase domains [95]. The HER family of tyrosine kinase receptors includes four members: HER1/epidermal growth factor receptor (EGFR), HER2, HER3 and HER4 that are expressed on tumor cell surfaces to mediate cellular growth and survival signals [96] during interactions with their ligands in the tumor microenvironment [97,98]. Exosomes secreted by HER-over-expressing cancers, including breast [99-101], pancreatic [102], brain [103,104], and gastric cancer [105], have been shown to display HER proteins from their native tumors. For example, in HER2 over-expressing breast cancer, an aggressive form of disease that accounts for 25 % of all breast cancers [106], exosomes display the HER2 oncoprotein on their surfaces [99-101]. Cancers that exhibit HER-dependent growth have also been reported to release exosomes that display EGFR ligands, including amphiregulin [107], TGF-α [107], heparin-binding EGF-like growth factor (HB-EGF)[107], EGFR [108] and the truncated and constitutively active form of EGFR, variant III (EGFRvIII), which causes unregulated growth of cancer cells [104]. Display of HER family members and their ligands on exosomes facilitates the spread of growth-stimulating and metastatic signals to several types of target cells. In a study by Al-Nedawi et al. [104], microvesicles derived from glioma cells transferred EGFRvIII to receptor-null glioma cells to promote mitogenesis, pro-survival signaling, and expression of VEGF [104]. EGFR from tumor-derived microvesicles can also be transferred to endothelial cells, eliciting VEGF upregulation and tumor angiogenesis [108]. In another study, exosomes from breast cancer and colorectal tumors displayed amphiregulin on their surfaces, which engaged with HER1/EGFR on tumor cells to increase their invasiveness [107]. Significantly, exosomal amphiregulin was found to be 5 times more efficient at increasing tumor invasiveness compared to the same concentration of soluble recombinant amphiregulin [107]. These data point toward tumor-derived exosomes as being major purveyors of oncogenic signals between cells.

**Figure 2:**
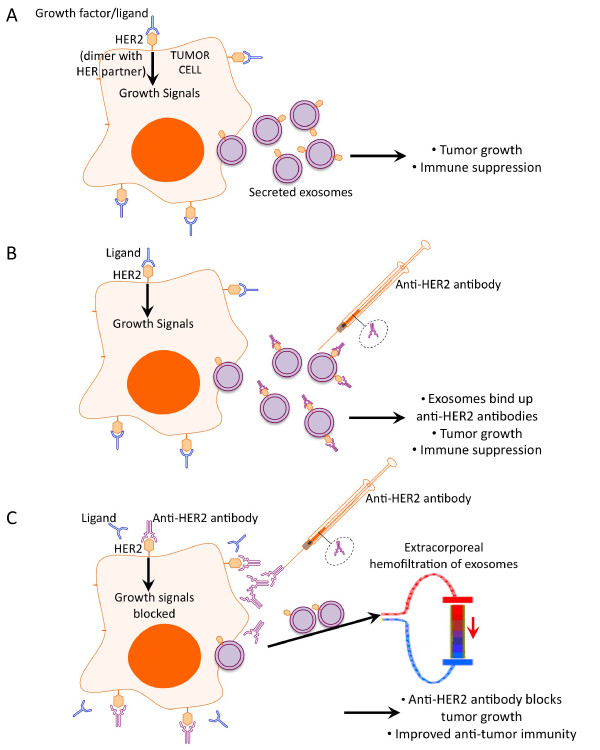
Proposed effects of exosome depletion on the activity of therapeutic antibodies in cancer. **(A)** Tumor-secreted exosomes display oncoproteins from their originating tumor cell. This example depicts HER2 over-expressing tumor cells releasing HER2+ exosomes that promote tumor growth and immune suppression, as described in [99-101]. **(B)** Monoclonal antibodies administered for immunotherapy can be sequestered by tumor-derived exosomes, owing to the display of oncogenic proteins on the exosomal surfaces [99-101,114]. In this example, HER2+ exosomes bind to anti-HER2 antibodies (for example, Herceptin®) and limit the bioavailability of antibodies. Consequently, continued tumor growth is permitted via interactions between HER proteins on the surfaces of tumor cells (consisting of dimers of HER2 with another HER family member), and growth factors/EGFR ligands in the tumor microenvironment. **(C)** A strategy for therapeutic filtration of exosomes from the circulation (shown here) or pharmacological methods of targeting exosome release by cancer cells could enhance the efficacy of immunotherapy. Conceptually, removal of exosomes from the bloodstream would allow therapeutic anti-HER2 antibodies to block HER-related signaling on tumor cells, thereby also alleviating exosome-mediated immune suppression and other pro-cancer activities.

An important pro-cancer effect of cancer exosomes is in mediating resistance to immunotherapeutic agents. The humanized monoclonal antibody Herceptin® (trastuzumab; Genentech Inc., San Francisco, CA), which binds to the extracellular domain of HER2, is the standard of care for breast cancers with HER2 amplification. Herceptin® binds to HER2 with high affinity and evokes a broad range of anti-tumor effects including direct inhibition of HER signaling, induction of antibody-dependent cell cytotoxicity (ADCC) by NK cells and possibly through downregulation (internalization) of HER proteins [109]. HER2 displayed on the surfaces of breast cancer exosomes has been shown to bind and sequester the therapeutic monoclonal antibody Herceptin®, thereby allowing continued tumor cell proliferation [101]. This decoy effect of breast cancer exosomes also shields target cells from ADCC mediated by NK cells [100]. The observation that advancing cancer is associated with increased exosome secretion by tumors as well as increased exosome binding to Herceptin® suggests that exosomes permit cancer progression and metastasis by limiting drug availability [101]. Indeed, exosome secretion in HER2 over-expressing breast cancer could be a contributing factor to the fact that the overwhelming majority of breast tumors become refractory to treatments directed at HER2 [110-113]. The schematic in Figure 2 depicts the roles of cancer exosomes in resistance to monoclonal antibody therapy, illustrating exosomes in HER2 over-expressing cancer as an example.

A second example of exosome-mediated resistance to monoclonal antibody therapy is observed in B cell lymphoma. CD20-bearing tumor exosomes have been demonstrated to bind to and intercept anti-CD20 antibodies (i.e. the therapeutic antibody rituximab) and also consume complement, thereby impairing ADCC and complement-dependent cytolysis against tumors [114]. Strikingly, in patients undergoing treatment for B cell lymphoma, approximately one third to one half of the plasma rituximab is bound to exosomes three hours following administration of the therapeutic antibody [114]. Removal of exosomes from plasma samples resulted in significant improvements in the cytolytic activity of rituximab against tumor cell lines and against autologous tumor cells *in vitro.* These data suggest that a strategy for targeting exosomes could be beneficial for unmasking the efficacy of therapeutic antibodies.

In addition to interfering with the activity of immunotherapeutic agents, tumor-derived exosomes also participate in the resistance of tumors to certain chemotherapy drugs. A role of exosomes in drug export from tumor cells was suggested by observations that cisplatin-resistant ovarian cancer cells displayed reduced lysosomal content of platinum and increased secretion of exosomes containing platinum as compared to cisplatin-sensitive cells [115]. Similarly, cisplatin removal by melanoma cells occurs via secretion of intracellular organelles called melanosomes, thereby impairing the drug’s localization to the nucleus [116]. In a study by Shedden et al. [117], the chemosensitivity profiles of NCI’s panel of 60 cancer cell lines were inversely correlated with expression of genes related to vesicle secretion. Accordingly, intra-vesicular accumulation of the therapeutic agent doxorubicin was associated with high rates of vesicle shedding by chemoresistant cells [117]. Based on these observations, the idea has been raised that drugs that interfere with microtubule stability, such as taxanes and vinca alkaloids, could serve as inhibitors of exosome secretion [118]. Although these drugs are already used for treating specific cancers, the cytotoxicity of these agents would hinder their applicability as additive therapies for ameliorating tumor-derived exosomes in cancer patients. Accordingly, other means for modulating exosomes are being explored, such as methods for altering the composition of exosomal proteins that promote malignancy [118]. For example, the dietary polyphenol curcumin reduces the immune suppressive activities of breast cancer exosomes against NK cells, which is believed to occur due to alterations in ubiquitination of proteins during sorting of cargo into ILV [119].

A promising alternative for inhibiting exosome secretion involves targeting vacuolar H^+^-ATPase-driven efflux pumps using proton pump inhibitors (PPIs), which are widely prescribed for suppressing gastric acid [120]. Since the activity of PPIs depends on acidic conditions, these agents should exhibit a degree of selectivity for tumors without introducing toxicity [1]. Vacuolar H^+^-ATPases are overactive in tumors, pumping high concentrations of protons across the plasma membrane to generate highly acidic extracellular microenvironments [120,122]. PPIs disrupt these pH gradients, leading to intracellular acidification and death of cancer cells [121]. Significantly, PPIs have been demonstrated to impair the release of acidic vesicles by cancer cells, thereby increasing the cytoplasmic retention of cytotoxic drugs and sensitizing tumors to chemotherapeutic agents [120]. In one study of three mouse tumor models, inhibition of exosome secretion using dimethyl amiloride, an inhibitor of H^+^/Na^+^ and Na^+^/Ca^2+^ channels, was effective for mitigating the immune suppressive effects of exosomes and restoring the responsiveness of cancer-bearing hosts to the chemotherapeutic agent cyclophosphamide [76]. The DMA analog amiloride, which also inhibits exosome release, was shown to decrease the immune suppressive activity of serum from 11 patients with colorectal cancer who were receiving this agent for hypertension [76]. Another possible option for targeting exosome secretion involves using sphingomyelinase inhibitors. Indeed, exosomes are enriched for ceramide, which is generated through the activity of sphingomyelinases and is involved in sorting of endosomal proteins into MVB [123]. Hence, the diverse pharmacological approaches for inhibiting exosome secretion should be investigated further and compared for their *in vivo* efficacy at unmasking immune function and therapeutic responses in cancer.

## Extracorporeal Hemofiltration of Circulating Factors as a Therapeutic Strategy in Cancer

Another promising cancer treatment strategy involves extracorporeal hemofiltration of immune suppressive factors including exosomes from the circulation. In a pioneering study by Dr. Rigdon Lentz, continuous whole blood ultrapheresis was used to remove low molecular weight proteins (<120, 000 daltons molecular weight) from the blood of 16 cancer patients of which 6 patients presented with a minimal 50 % reduction in the sizes of their tumors [124]. The primary targets were considered to be serum cytokine receptors that impede anti-neoplastic immune responses [125] since exosomes and their roles in cancer were not appreciated at that time. Clinical approval was also granted for application of the Prosorba Column, known as “Protein-A Immunoadsorption Therapy”. This plasma filtering device consists of highly purified protein A from *Staphylococcus aureus* covalently linked to a silica matrix to capture circulating immunoglobulin G (IgG) and immune complexes containing IgG, which was FDA-approved for rheumatoid arthritis and idiopathic thrombocytopenic purpura as a complementary therapy for clearing pathogenic autoantibodies. In a study examining the efficacy of the Prosorba column in cancer, there was a measurable reduction in tumor burden in 22 of 104 patients and increased immune system activity reportedly occurred in the hours following treatment [126,127]. However, in a Phase II trial of metastatic breast cancer, circulating immune complexes were not detected in the majority of patients and treatment with the Prosorba column did not confer clinical benefits [128].

**Figure 3:**
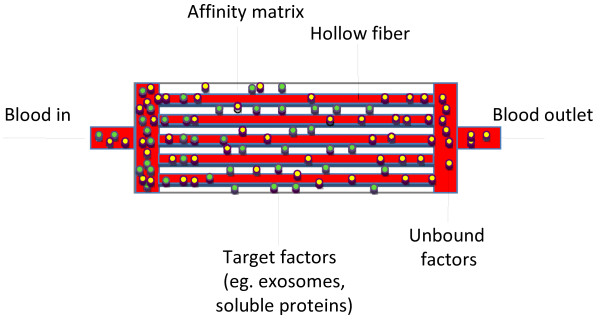
**Schematic of Aethlon’s ADAPT™ device platform.** This technology consists of plasmapheresis cartridges that allows blood cells to pass through the hollow fibers while serum components < 200 nm in size fit through the hollow fiber pores to interact with the affinity matrix. The matrices can be customized with one or more affinity substrates comprising monoclonal antibodies, lectins, aptamers or other affinity agents to specifically capture and remove tumor-derived exosomes and other soluble oncoproteins from the bloodstream using kidney dialysis or CRRT units.

Given the recent appreciation for the roles of exosomes as malignancy-associated factors, an extracorporeal strategy for specifically targeting exosomes is an attractive therapeutic option for cancer. Aethlon Medical has devised a therapeutic hemofiltration approach, termed the Aethlon ADAPT™(adaptive dialysis-like affinity platform technology) system. This technology consists of immobilized affinity agents in the outer-capillary space of hollow-fiber plasma separator cartridges that integrate into standard dialysis units or continuous renal replacement therapy (CRRT) machines. As the patient’s blood passes through device, plasma components < 200 nm in size travel through the porous fibers and interact with the immobilized affinity agent(s) to which target molecules are selectively adsorbed while blood cells and non-bound serum components pass through the device (Figure 3). This device strategy is versatile, owing to the fact that innumerable antibodies and other affinity reagents, such as aptamers and protein ligands, can be incorporated into the cartridges for capturing single or multiple targets. Although ADAPT™ therapies require that patients undergo a surgical procedure for vascular access, this subtractive strategy for addressing cancer exosomes would not introduce drug toxicity or interactions risks, thereby offering an advantage over pharmacological approaches. Hence, this device strategy offers an approach for targeting exosomes that should be examined for its utility as an adjunct therapeutic candidate to standard of care cancer treatments.

There is a clinical precedent that supports the safety and efficacy of affinity hemodialysis using ADAPT™ devices. The first ADAPT™ device, the Hemopurifier®, consists of a plasmapheresis cartridge to which the lectin *Galanthus nivalis* agglutinin (GNA) is covalently coupled to capture viruses on the basis of the high mannose glycoproteins on viral envelopes [129]. Aethlon has conducted clinical studies of patients infected with hepatitis C virus (HCV) that were treated with the Hemopurifier® inserted into standard dialysis extracorporeal circuits for up to 3 times weekly for 4–6 hours/treatment. Of the approximately 100 treatment experiences with the Hemopurifier® thus far, this therapy was well tolerated and the frequencies of device-related adverse events were within the range of those occurring during routine dialysis (data not shown). Hemopurifier® therapy reduced the viral load in HCV-infected patients who were not concurrently receiving anti-viral drugs, and had a remarkable impact in improving patient responses to ribarvirin and pegylated interferon therapy ([129] and data not shown).

Tumor-derived exosomes are enriched for high mannose structures on their surface glycoproteins [130] and have been demonstrated to bind to lectins, including GNA ([131] and our unpublished observations). Given the similarity in size and surface topology between virions and cancer exosomes [132], the Hemopurifier® is currently being evaluated for its efficacy for capturing exosomes secreted by tumor cell lines and present in biologic fluids from cancer patients. Since ADAPT™ devices implementing antibodies as affinity substrates have been constructed for other indications [133,134], an antibody-based approach could similarly be utilized for recognizing tumor-specific proteins on exosomal surfaces in order to capture cancer exosomes while sparing exosomes produced by non-malignant cells. For example, in HER2 over-expressing breast cancer, anti-HER2 antibodies could be utilized to remove HER2 expressing exosomes as well as soluble HER2, which is proteolytically cleaved from the cancer cell surface and also neutralizes the activity of Herceptin® [135]. Several studies reveal that high levels of shed HER2 are associated with high-grade tumors, lymph node metastasis, and higher mortality of breast cancer patients [136-138]. Thus, the capability for simultaneously removing both soluble and exosome associated oncoproteins using the ADAPT™ system could offer a unique strategy for improving the therapeutic outcomes for cancer patients.

In a therapeutic context, the fact that the ADAPT™ system can access soluble factors in the circulation but not those within the tumor or regional lymph nodes makes this device strategy suitable for metastatic cancers. Indeed, tumor-derived exosomes have been demonstrated to transport molecular signals involved in angiogenesis and stroma remodeling for tumor cell adhesion and growth during priming of the pre-metastatic niche [139,140]. Moreover, since exosome production is determined by tumor size and growth rate [16,17], the duration and frequency of ADAPT™ therapy would require optimizing in order to achieve a clinically beneficial level of exosome depletion from the circulation. The device strategy could also be tailored for different types/stages of cancer and using devices incorporating different affinity agent(s). Currently, although a spectrum of biologic effects of cancer exosomes have been identified *in vitro* and in experimental animals, the impact of diminishing exosomes therapeutically must still be studied in terms of the potential efficacy in promoting immune recovery and hindering tumor growth in a clinical setting.

## Conclusions

Exosomes have emerged as being important vehicles for intercellular communication and for modulating immune responses, owing to their content of proteins and genetic material that mirror their cells of origin. Whereas exosomes from activated lymphocytes can possess immune stimulatory functions, there are many physiologic examples of exosomes exerting tolerogenic functions during dampening of immune responses, oral tolerance and pregnancy. In cancer, the tolerogenic activities of exosomes represent pathological responses whereby tumor cells secrete vast amounts of immune inhibitory exosomes that hinder anti-cancer immune responses. Tumor-derived exosomes are involved in the fundamental aspects of cancer pathogenesis including growth, metastasis, angiogenesis, and immune suppression. Therefore, to address the unmet need for a strategy to target tumor-secreted exosomes, one possible option involves a therapeutic hemofiltration approach, the Aethlon ADAPT™ system, which is designed to selectively capture and remove target particles such as exosomes from the entire circulatory system. This technology consists of hollow fiber plasma filtration cartridges constructed with affinity agents that are fitted for existing dialysis machines. The ADAPT™ system has the potential to address a variety of types and stages of cancer since it can incorporate diverse affinity agents for capturing cancer-specific exosomes on the basis of their display of surface proteins (using antibodies) and/or glycoproteins (using lectin affinity agents). The emerging evidence that tumor-secreted exosomes are involved in mediating resistance to therapies provides an impetus for exploration novel therapeutic options for addressing the immune inhibitory and tumor growth-promoting effects of cancer exosomes.

## Abbreviations

ADAPT, (adaptive dialysis-like affinity platform); ADCC, (antibody dependent cell cytotoxicity); CRRT, (Continuous renal replacement therapy); DC, (Dendritic cells); DMA, (Dimethyl amiloride); EGFR, (Epidermal growth factor receptor); GNA, (Galanthus nivalis agglutinin); HCV, (Hepatitis C virus); HER, (Human epidermal receptor); ILV, (Intralumenal vesicles); MICA/B, (MHC class I chain-related proteins A and B); MIIC, (Major histocompatibility complex class II-enriched compartment); MDSC, (Myeloid-derived suppressor cells); MVB, (Multivesicular bodies); PPIs, (Proton pump inhibitors); Treg, (T regulatory); UL-BP, (UL-16 binding proteins).

## Competing interests

JAJ, RHT and AMM are employees and/or shareholders of Aethlon Medical. CSC has no competing interests.

## Authors’ contributions

JAJ and RHT conceived of the ADAPT™ technology described in this manuscript. AMM drafted the manuscript with the participation of CSC, JAJ, and RHT. All authors read and approved the final manuscript.
